# Production of biodiesel from waste fish fat through ultrasound-assisted transesterification using petro-diesel as cosolvent and optimization of process parameters using response surface methodology

**DOI:** 10.1007/s11356-024-32702-6

**Published:** 2024-03-13

**Authors:** Soumya Parida, Harveer Singh Pali, Anurag Chaturvedi, Abhishek Sharma, Dhinesh Balasubramanian, Ravikumar Ramegouda, Viet Dung Tran, Van Giao Nguyen, Femilda Josephin Joseph Shobanabai, Edwin Geo Varuvel

**Affiliations:** 1 G.L.Bajaj Institute of Technology and Management, 201306, Greater Noida, India; 2 Renewable Energy and Alternative Fuel Lab, National Institute of Technology, Srinagar-190006, Jammu-Kashmir, India; 3 Department of Mechanical Engineering, Loknayak Jayprakash Institute of Technology, Chapra-841302, Bihar, India; 4 Department of Mechanical Engineering, Mepco Schlenk Engineering College, Sivakasi, Tamil Nadu India; 5 Department of Mechanical and Automobile Engineering, CHRIST University, Bangalore, India; 6 PATET Research Group, Ho Chi Minh City University of Transport, Ho Chi Minh City, Vietnam; 7 Institute of Engineering, HUTECH Universit, Ho Chi Minh City, Vietnam; 8 Department of Computer Engineering, Faculty of Engineering and Natural Sciences, Istinye University, Istanbul, Turkey; 9 Department of Autotronics, Institute of Automobile Engineering, Saveetha School of Engineering, Saveetha Institute of Medical and Technical Sciences, Chennai, 602105 Tamil Nadu India; 10 Department of Mechanical Engineering, Faculty of Engineering and Natural Sciences, Istinye University, Istanbul, Turkey; 11 Department of Automobile Engineering, Faculty of Engineering and Technology, SRM Institute of Science and Technology, Kattankulathur, Tamil Nadu 603203 India

**Keywords:** Homogeneous catalyst, Free fatty acid, Cosolvent, Fourier transform infrared spectroscopy, Optimization, Ultrasonication

## Abstract

Biodiesel is a highly promising and viable alternative to fossil-based diesel that also addresses the urgent need for effective waste management. It can be synthesized by the chemical modification of triglycerides sourced from vegetable origin, animal fat, or algal oil. The transesterification reaction is the preferred method of producing biodiesel. However, the non-miscibility of alcohol and oil layer causes excessive utilization of alcohol, catalyst, and a substantial reacting time and temperature. In the current investigation, transesterification of waste fish oil was performed with petro-diesel as cosolvent, under the influence of ultrasound energy. The combination of both techniques is a unique and efficient way to minimize the mass transfer limitations considerably and hence reduces the parameters of the reaction. It is also a sincere effort to comply with the principles of green chemistry. The optimum reaction conditions were obtained using response surface methodology (RSM) that were as follows: molar ratio of methanol to oil 9.09:1, catalyst concentration of 0.97 wt%, cosolvent concentration of 29.1 wt%, temperature 60.1℃, and a reacting time 30 min. Under these listed conditions, 98.1% biodiesel was achievable, which was in close agreement with the expected result. In addition, the cosolvent removal step from the crude biodiesel was also eliminated as it could be employed as a blended fuel in CI engines.

## Introduction

Excessive dependence on fossil fuels has led to its expeditious depletion, soaring prices, and addition to environmental woes by emitting more gases causing the greenhouse effect. Hence, the discovery of various alternate renewable sources of energy is the need of the day that could address the abovementioned concerns. Biodiesel, obtained from biomass, acts as a convincing alternative to conventional diesel derived from petroleum (Edwin Geo et al. 2009). It is more environmentally friendly compared to its petroleum counterpart. The most commonly preferred method of preparing biodiesel industrially is the transesterification of oils (vegetable oil, animal fats, or algal oil), in which triglyceride present in the oil is reacted with a lower alcohol in the presence of an acidic or an alkaline catalyst (Thiruvenkatachari et al. 2021). This practice is prevalent and well-recognized (Venugopal et al. 2021). Although biodiesel stands superior to petro-diesel in many aspects like its emission profile, source of origin, etc., its cost stands as a hurdle in its widespread commercialization. Currently, biodiesel costs more than petro-diesel and the major causes behind it are the expensive feedstock and the high reaction parameters. The immiscibility of the alcohol and oil layers causes mass transfer limitations that lead to excessive consumption of alcohol, catalysts, and increased energy requirements. The traditional method to overcome the difficulty of mass transfer is mechanical stirring which takes a long time for reaction as well as product separation. However, some process intensification techniques like membrane reactors hydrodynamic cavitation, ultrasonic cavitation, and microwave irradiation have proven to accelerate the rate of transesterification dramatically. These methods require comparatively low reagents and less reaction time, and hence much energy can also be saved (Duraisamy et al. 2023).

Both microwave- and ultrasound-assisted transesterification have proven to be effective process intensification methods. In many cases, microwave irradiation is found to be more effective than the ultrasound method. However, the ultrasound method can be operated at much lower temperatures than the microwave method (Sara et al. 2016). Moreover, the microwave method is uncertain from the environmental perspective, when used on a pilot scale, owing to the use of electromagnetic radiation. Some studies have also reported a combination of both methods for biodiesel synthesis (Gole and Gogate 2013; Hsiao et al. 2010; Kodgire et al. 2022). The supercritical process, on the other hand, is the most effective one in terms of chemical conversion, but the alcohol requires a high temperature and pressure resulting in considerable loss of fatty acid methyl ester (FAME) owing to its degradation reaction at such elevated temperatures (Sajjadi et al. 2014). The ultrasound process is environmentally benign, and has a very simple reactor showing high energy efficiency and a shortened reaction time and product separation (Oh et al. 2012). Ultrasound causes the formation of cavitation bubbles that grow larger and eventually burst violently, causing micro turbulence that breaks up the phase boundary (Tan et al. 2019). The bursting of cavitation bubbles also creates localized high-temperature areas along with many highly reactive species. This lowers the activation energy and accelerates the reaction rate. According to studies by Sharma et al., the activation energy of an ultrasound-assisted process is lowered approximately 1.5–1.7 times compared to the mechanically stirred method (Sharma et al. 2020). Therefore, using ultrasound energy to make biodiesel eliminates the need to stir the mixture and reduces the required temperature. Sáez-Bastante et al. (2014) reported that the expenditure for ultrasound energy-aided transesterification was lower compared to the conventional mechanical stirring-based transesterification due to reduced reaction inputs like methanol:oil molar ratio, reaction time, and energy. Several other studies have also confirmed the same observation (Carmona-Cabello et al. 2019; Oliveira et al. 2021).

Ultrasound-assisted homogeneous alkali-catalyzed transesterification is proven as one of the highly promising routes to manufacture biodiesel from feedstock oil with low free fatty acid (FFA) composition. Reduced reaction parameters and high biodiesel yield compared to the mechanically stirred process are reported (Gupta 2021; Suresh et al. 2021). Under optimal state (the molar ratio is 5:1, 0.7 wt% catalyst, and the duration of the reaction is 50 min), more than 99% of the canola oil was transformed into biodiesel by transesterification using ultrasonic irradiation (Thanh et al. 2010). With the help of the artificial neural network (ANN) model, researchers were able to identify the optimal conditions of this process for sunflower oil using ultrasonic energy. This ultimately led to a maximum FAME production of 89.9% with a methanol/oil molar ratio of 7.50:1, catalyst loading of 0.70%, and reacting temperature of 30 °C and time 60 min (Rajković et al. 2013). The production of biodiesel using discarded cottonseed cooking oil yielded a substantial quantity of product in a KOH-catalyzed ultrasound-assisted process, and the reaction parameters were determined to be a methanol/oil molar ratio of 6.1:1, concentration of catalyst 0.46 wt%, reacting temperature 53.2 °C, and maximum product yield of 97.76% (Ragavan and Roy 2011).

The addition of cosolvents like tetrahydrofuran, hexane, acetone, di-ethyl ether, and so on has also been proven to enhance the mass transfer. A cosolvent could homogenize the reactant phases, alcohol, and oil, without any other effect on the reactants (Hájek et al. 2020). When resistance to the transfer of mass between phases disappears, the transesterification reaction increases, and the rate constant’s value increases (Guan et al. 2009; Roosta and Sabzpooshan 2016). Consequently, there is a decrease in specific reaction parameters like the concentration of the catalyst, the temperature of the reaction, and the molar ratio of alcohol to oil. Fadhil et al. discovered that using cosolvent in the homogeneously catalyzed transesterification of fish oil decreased the methanol-to-oil molar ratio and reaction temperature (Fadhil et al. 2015). Alhassan et al. also found that the addition of cosolvent may reduce the duration of the reaction by 60% (Alhassan et al. 2014). They accelerate the process and do not affect the quantity of biodiesel produced (Parida 2021; Roosta and Sabzpooshan 2016). Usually, biodiesel is used to make cosolvents like hexane, acetone, ethyl acetate, tetrahydrofuran, dimethyl ether, tert-butyl methyl ether, etc. At 25 °C, with the addition of cosolvents, including dimethyl ether, diethyl ether, tert-butyl methyl ether, and tetrahydrofuran, one of the earlier experiments succeeded in producing biodiesel from sunflower oil. The results proved that adding a cosolvent increases the reaction rate since almost all reactions were done in 20 min. On the other hand, there was only a 78% conversion rate when cosolvent was not present (Hassan et al. 2020). The effect of cosolvent on transesterification has also been looked into for systems with different catalysts (Chumuang and Punsuvon 2017; Laskar et al. 2020). With the help of a CaO catalyst made from waste biomass and acetone, used fish oil was turned into biodiesel. The best conditions for the reaction were 20 wt% of acetone loading, 2 h of reaction time, 3 wt% of catalyst loading, and a 6:1 methanol-to-oil molar ratio. This gave a 98% yield of biodiesel. Biodiesel was synthesized from spent cooking oil using a heterogeneous catalyst, namely kettle limescale, and cosolvents hexane and tetrahydrofuran (THF) (Aghel et al. 2018). Under the optimum conditions of reaction of biodiesel yield in the presence of n-hexane, it was reported as 97.03% and 95.21% with THF. In the same process, refined coconut oil with OH-impregnated CaO as a heterogeneous catalyst, exhibited 66.36 and 81.7% conversion, without and with the addition of cosolvent respectively (Bambase et al. 2021). In a contradictory study, heterogeneously catalyzed transesterification yielded a lesser amount of ester with cosolvent compared to the one without it. It was attributed to either the higher dilution of reactants upon the addition of cosolvents or the cosolvents blocking the active catalyst sites (Hájek et al. 2020). Several investigations used a combination of cosolvent with other process-intensifying technologies like ultrasound energy, microwave irradiation, hydrodynamic cavitation, and supercritical methanol (Erchamo et al. 2021; Farvardin et al. 2022; Martínez et al. 2017).

Transesterification of sunflower oil in the presence of the cosolvent assisted by hydrodynamic cavitation (Nikolić et al. 2022) resulted in further enhancement in the product output with minimized reaction requirements. Despite all the positive results associated with cosolvent-aided transesterification, the method cannot be considered completely environment-friendly and sustainable. The reason was the removal of the toxic organic cosolvents from the crude biodiesel that needed further energy (Gholami et al. 2021). Environmentally benign crude biodiesel was used as cosolvent, to investigate the transesterification of sunflower oil using CaO as a catalyst (Todorović et al. 2019). The choice of cosolvent eliminated the step of removing the cosolvent.

In the present study, the researchers investigated the homogeneous alkali-catalyzed transesterification of discarded fish oil with petro-diesel as a cosolvent and ultrasound-assisted method for process intensification. The study was done to devise an environmentally friendly and economical method of biodiesel production. The study was done to devise an environmentally friendly and cost-effective method of biodiesel production. The use of petro-diesel as a cosolvent could be considered sustainable because it was not required to be removed from crude biodiesel as it could be directly used in the form of blended fuel. Reactions under the influence of ultrasound energy (Hoang 2021; Rudzki et al. 2022) have proven to be energy- as well as reagent-saving. The use of waste fish oil as feedstock also adds valorization to an otherwise discarded waste.

The coastal regions consuming huge quantities of fish face a constant problem in getting rid of fish waste and dead fish. Valorization of these fish wastes is an excellent way of getting rid of them along with the production of value-added products like biodiesel.

However, several studies have reported that the viscosity and acidity of biodiesel derived from fish waste and other oils are much greater than those of conventional diesel (Jayasinghe and Hawboldt 2012), resulting in performance and oxides of nitrogen (NOx) issues in diesel engines (Thiyagarajan et al. 2018; Jayasinghe and Hawboldt 2012). Some studies even suggested that the acidic value depended remarkably on the parts of the fish used, such as viscera, skin, and muscles. It also depended on the fish species (Karkal and Kudre 2020). Studies on salmon oil having high acid value suggested that one-step alkaline-catalyzed transesterification was not a very efficient way to produce biodiesel from it (Devarajan et al. 2022), indicating this process needs to be modified (Santos et al. 2015).

As mentioned previously, the present investigation focused on the alkali-catalyzed transesterification of waste fish oil using ultrasound energy and petro-diesel as a cosolvent. The study employed response surface methodology (RSM) and a central composite design (CCD) to analyze the impact of reaction time, ratio of alcohol and oil, concentration of catalyst, and cosolvent concentration on the production of the desired product.

The primary objective was to know the impact of the process variables on the FAME (fatty acid methyl ester) content and identify the ideal parameters for achieving the maximum FAME content. Notably, this research represents the first documented instance of statistically optimizing the homogeneously catalyzed ultrasound-assisted transesterification process while utilizing petro-diesel as a cosolvent.

## Materials and methods

### Materials

Waste fish fat was collected from a local wholesale fish market in Machilipatnam market, Andhra Pradesh. Anhydrous methanol (99.5%) and sodium hydroxide (NaOH) were purchased from M/s Finar, Ahmedabad. Petro-diesel was bought from a nearby BPCL (Bharat Petroleum Corporation Limited) oil filling station. The reference standard solution, methyl heptadecanoate, used to test FAME was purchased from Sigma-Aldrich. The ultrasonic processor was bought from M/s Oscar Ultrasonics Pvt. Ltd., in Mumbai, India. It has a frequency of 20 kHz and a maximum power output of 1 KW.

### Methods

**Fig. 1:**
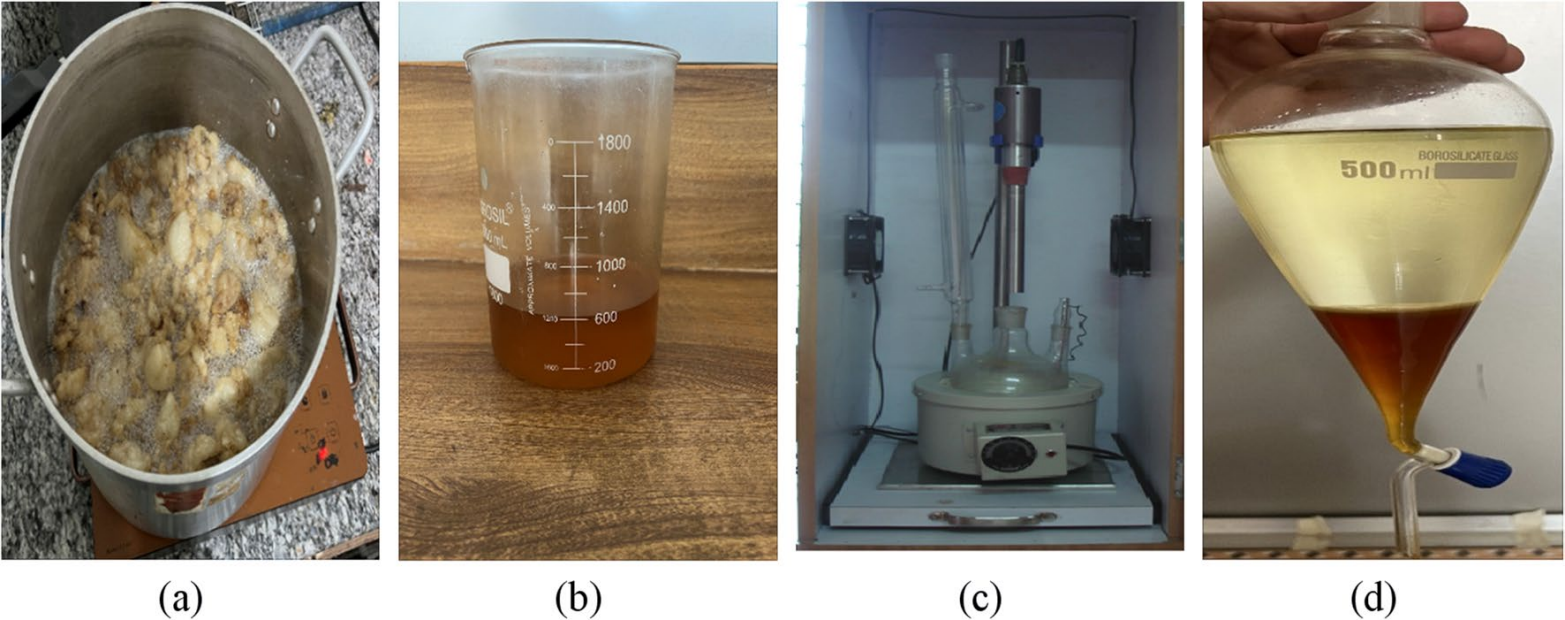
Flow diagram of ultrasonic biodiesel production. **a** Heating of fish fat, **b** filtered fish oil, **c** experimental setup of batch type ultrasound reactor, **d** biodiesel separation in separating flask

Fish oil extracted from fish fat was directly subjected to homogeneously catalyzed transesterification. The ultrasound reactor comprised a reaction vessel (three-necked round bottom flask), a heating mantle, and an ultrasonic probe and horn. The sonicator has a maximum power output of 1 kW and an amplitude ranging from 25 to 100%. Figure 1 shows the stepwise preparation of biodiesel from waste fish fat using an ultrasonic reactor. The reaction was conducted at a constant frequency of 20-kHz frequency and 50% amplitude. One hundred grams of accurately weighed fish oil was taken in the reaction vessel and heated to 10 degrees below the required reaction temperature (Balasubramanian 2022; Hoang et al. 2021). This was because the bursting of cavitation bubbles caused a temperature rise. A calculated quantity of sodium hydroxide solution (KOH) made in methanol (MeOH) was added gradually, followed by the addition of cosolvent petro-diesel (Balasubramanian 2019). The ultrasonic horn was dipped 5 mm into the reaction mixture, and sonication was done in pulse mode (2 s on and 2 s off) to save energy. The reaction was conducted for 45 min, and the temperature was maintained constant throughout the reaction by turning the heater on and off intermittently (ultrasonication raises the temperature of the reaction mixture). The reaction mixture samples were drawn periodically to monitor the progress of the reaction. After the stipulated reaction time, the products were put into a separating funnel. Two layers are formed: the top is a mixture of biodiesel and diesel, and the bottom comprises the byproduct glycerol. The biodiesel and petro-diesel mixture were separated, cleaned, and tested. Table 1 shows some of the most important physical traits.

**Table 1:** Comparative analysis of the fish waste biodiesel compared with petro-diesel

Property	Unit	ASTM method	Biodiesel	Petro-diesel	Equipment used	Uncertainty (%)
Density (at 35℃)	g/cc	D1448	0.862 to 0.875	0.835	Density meter DMA-450	0.1%
Kinematic viscosity (at 40℃)	cSt	D445	2.0 to 5.1	3.1	Antonpar viscometer	0.3%
Calorific value (CV)	MJ/kg	D6751	39.5 to 42	43.5	Parr bomb calorimeter 6100	0.1%
Acid value	mg KOH/g	D664	0.1 to 0.2	0.31	Potentiometer titrator	0.1%
Flash point	°C	D93	110 to 138	66	Pensky-martens flash point	0.05%
Cold filter plugging point (CFPP)	°C	D6371	− 4 to − 1	− 14	Cold filter plugging point–automatic NTL 450	0.1%

**Fig. 2:**
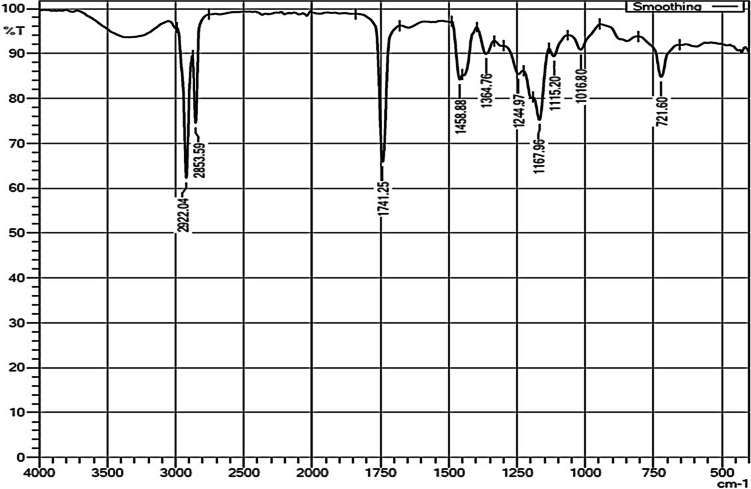
FTIR analysis of Fish biodiesel

Fourier transform infrared spectroscopy (FTIR) was used to determine the numerous functional groups in the fuel sample. In Fig. 2, the FTIR graph represented spectra of distinct peaks of aliphatic compounds, specifically alkanes (methyl group in ester) C–H stretching 2922 cm^−1^ and 2853 cm^−1^ and carbonyl ester group C = O stretching 1740 cm^−1^ and 1245 to 1115 stretching vibrations represented –C–O ester group. This observation indicated the conversion of triglyceride into methyl ester (biodiesel).

### Methodology

RSM is a mathematical technique that can be used to model and examine various issues where several variables influence the response, and the ultimate goal is to get the optimal outcome. Making a response surface to choose the sample point to make the ideal model without doing several tests is essential. Experiment design can be used to find these fitting points. The test matrix for this investigation was made using a complete factorial modeling method. The quadratic response surface models were made based on the effects of the experiments on the response variables, which were taken from the maximum factorial design matrix. The least square method is used to figure out relationships. In RSM, the input value is always inserted as a numerical value.

1 $$y=f\left({x}_{1},{x}_{2},{x}_{3},{x}_{4},\dots \dots \dots {x}_{n}\right)+\varepsilon$$

where ${x}_{1},{x}_{2},{x}_{3},{x}_{4},\dots \dots \dots {x}_{n}$ are the variables that go into the transesterification reaction, *y* is the amount of the product made, and $\varepsilon$ is the error.

The first step in response methodology is to find a good relation between the response and the independent variables. In general, partnership is defined by a second model, which can be explained as follows:

2 $${Y=\beta }_{o}+{\sum }_{i=1}^{k}{\beta }_{i}{x}_{i}+{\sum }_{i=1}^{k}{\beta }_{ii}{x}_{i}^{2}+{\sum }_{j\ge 1}^{k}{\beta }_{ij}{x}_{i}{x}_{j}+\varepsilon$$

In the above equation, *Y* represents the response; ${x}_{i}$ is the size of the factor; ${\beta }_{o}$, ${\beta }_{i},{\beta }_{ii}, \mathrm{and }{\beta }_{ij}$ are the coefficients of regression; $i \mathrm{and }j$ denote linear and quadratic coefficients; and the term $\varepsilon$ represents the experimental error. In this RSM model, the input variables are the reaction temperature, the catalyst concentration, the ratio of MeOH to oil, the reaction time, and the cosolvent. These variables could affect the output response: the amount of biodiesel made. Table 2 shows how much of each input factor is used. In this case, the midpoint is set to 0 for input variables. The CCD is given a value of − 2, − 1, 0, 1, or 2.

**Table 2:** The coded levels of input variables

Input factors	Coded levels				
	− 2	− 1	0	1	2
Temperature	50	55	60	65	70
Catalyst concentration (wt%)	0.25	0.5	0.75	1	1.25
MeOH:oil molar ratio	3:1	5:1	7:1	9:1	12:1
Time (minutes)	10	20	30	40	50
Cosolvent	0	10	20	30	40

### RSM modeling

The combination of control factors that lead to the most biodiesel production was examined for the study. The study of the variance factor was applied to the different parameters of the inputs to figure out the value of each parameter within the set range Anh Tuan Hoang (2021). The software used a linear regression model to determine how to get these results. The tests are done for different combinations of factors in the order shown in Table 3, which is based on the design matrix run order. The response values for each test are recorded. This includes at least 33 tests with different combinations of input parameters. Derringer’s RSM’s Desirability method helps with optimization. In this method, the best solution is the one people want the most. The appropriate set of variables for the engine’s functioning is the collection of variables that leads to the optimal outcome. Analysis of variance (ANOVA) gives real-world information about the benefits of the future. Table 4 displays the ANOVA results for yield. The *p*-value is considered a significant factor based on various research. Its value should not exceed 0.05 for multiple parameters. All the *p*-values above 0.05 are not considered, but if its value is less than 0.05, it can considerably affect the model. A computational method using the decision coefficient *R*^2^ has also been used to test the models. *R*^2^ values below 1 are thought to mean that the results of the experiment match the findings of the model. This means that it has high dependability. For making biodiesel, yield *R*^2^ is 98.57%, which is very close to 1. This shows that this model gives accurate results.

**Table 3:** Layout of the experimental matrix

	Inputs					Output
S. no	Temp. (^0^C)	CC (wt%)	MeOH:oil	Time (min)	Cosolvent (%)	Yield (%)
1	65	1	9	20	10	82
2	60	0.75	7	30	20	90
3	60	0.75	7	30	20	90
4	65	0.5	5	20	10	73
5	55	0.5	9	40	30	96
6	60	0.75	7	30	20	95
7	55	1	3	20	10	68
8	55	0.5	5	20	30	87
9	65	1	9	40	30	98.5
10	55	0.5	5	40	10	78
11	55	1	9	40	10	80
12	60	0.75	7	30	20	95
13	55	0.5	9	20	10	77
14	65	0.5	9	40	10	81
15	65	0.5	9	20	30	96
16	65	1	5	40	10	80
17	55	1	5	40	30	98
18	65	0.5	5	40	30	92
19	60	0.75	7	30	20	87
20	65	1	5	20	30	97
21	60	0.75	7	30	20	95
22	55	1	9	20	30	98
23	60	0.75	7	10	20	85
24	60	1.25	7	30	20	94
25	60	0.75	7	30	0	72
26	60	0.75	3	30	20	83
27	60	0.75	7	30	40	92
28	50	0.75	7	30	20	91
29	60	0.75	7	30	20	93
30	60	0.75	12	30	20	98
31	70	0.75	7	30	20	94
32	60	0.25	7	30	20	69
33	60	0.75	7	50	20	98

**Table 4:** The ANOVA results

		Production yield			
Sources	DF	Adj. SS	Adj. MS	*F*-value	*p*-value
Regression	5	1573.86	314.77	10.32	0.000
Temp (C)	1	16.94	16.94	0.56	0.043
CC (wt%)	1	216.75	216.75	7.11	0.013
MeOH:oil	1	293.36	293.36	9.61	0.004
Time (min)	1	129.66	129.66	4.25	0.049
Cosolvent	1	884.75	884.75	29.0	0.000
Lack-of-fit	21	762.8	36.32	3.58	0.050

### Optimization response

**Fig. 3:**
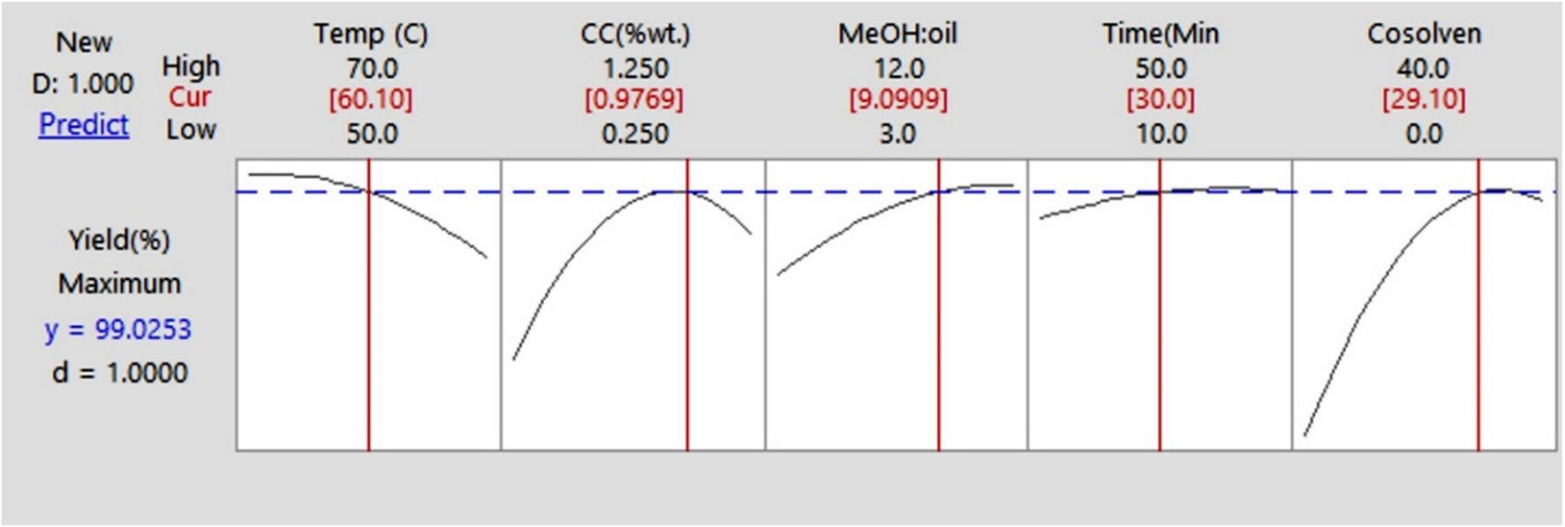
RSM optimizer

For the present analysis, the RSM optimizer (Fig. 3) is implemented to boost the operating parameters for biodiesel production.

A yield of 99.0253% was discovered when the reaction temperature was set to 60.1 when the concentration of catalyst was set to 0.9769% weight, when the ratio of MeOH to oil was 9.09, and when the reaction duration was set to 30 min with 29.1% cosolvent. A desirability strategy of one was used to accomplish this optimization. RSM may also estimate and alter the biodiesel reaction parameters in the test value series. This can be done by changing the values. It significantly reduces the time spent on research for studies, saving everyone time and money.

## Results and discussion

Transesterification is a reversible process involving a reaction between an oil and alcohol that are remotely miscible leading to excessive consumption of alcohol and catalyst. Hence, the amount of alcohol used and the concentration at which the catalyst is added affect the amount of biodiesel produced. The reaction time and reaction temperature also have a significant impact on the biodiesel or FAME yield. The link between the variables of the reaction and the FAME conversion may be shown graphically in response surface plots and contour graphs. Based on these findings, it was hypothesized that the maximum FAME conversion would be 99.0253% at a reaction temperature of 60.1, a catalyst concentration of 0.9769 wt%, a MeOH:oil ratio of 9.09, a reaction duration of 30 min, and a cosolvent concentration of 29.1% wt%, as can be seen in Fig. 2. This was in line with what the results of the experiment showed.

### Impact of molar ratio (MeOH:oil)

**Fig. 4:**
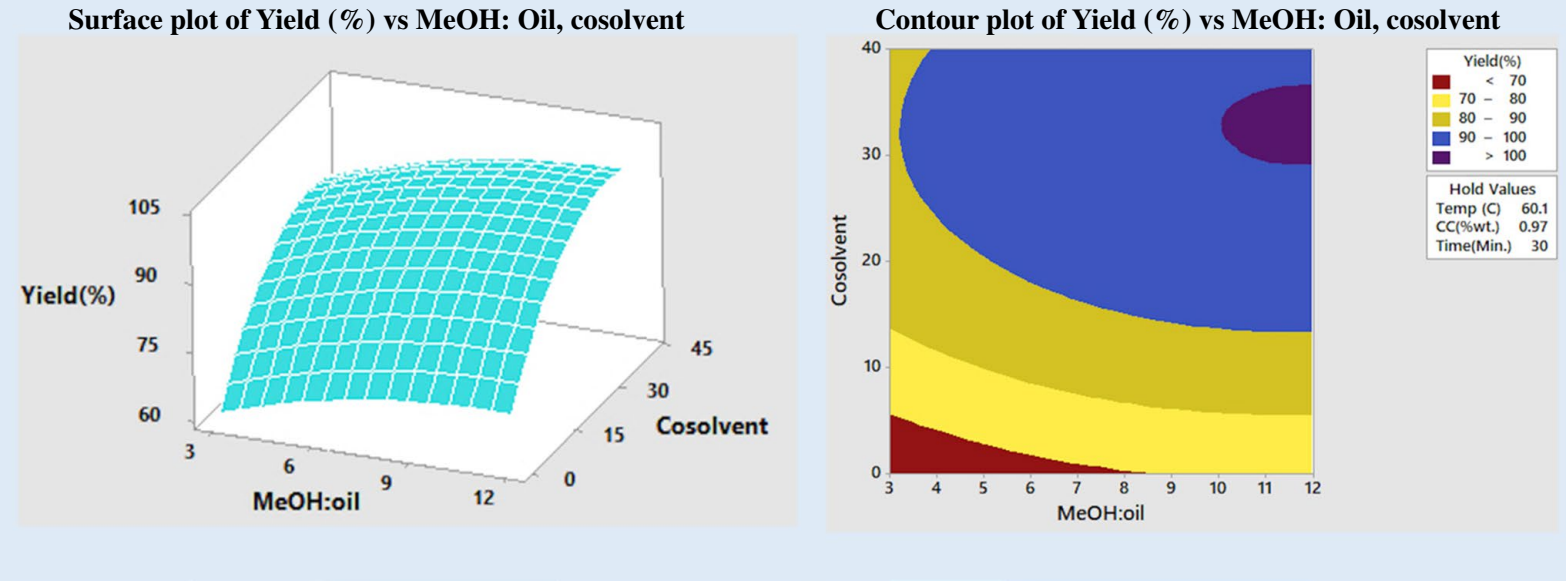
Impact of molar ratio and cosolvent on yield

Research on the effects of changing the methanol to oil molar ratio from 3:1 to 5:1, 7:1, 9:1, and 12:1 was conducted. Figure 4 is a response surface plot and contour map demonstrating the impact of the alcohol:oil molar ratio and cosolvent concentration on FAME production. All variables, including time, temperature, and booze content, are constant. The quantity of alcohol used was shown to affect the production of FAME. However, the boost was only discernible up to a 9:1 molar ratio. The output decreased when more alcohol was added. This may be because an excess of methanol promotes a reverse process that results in the recombination of glycerol and esters (Alsultan et al. 2021). For all alcohol-to-oil ratios, the inclusion of cosolvent significantly improved product yield. A product yield of 90% is possible even at the stoichiometric ratio of (3:1). Increases in both reaction rate and product yield may be attributed to the synergistic impact of ultrasonic energy and cosolvent in improving the miscibility of oil and alcohol, which is helpful for transesterification.

**Fig. 5:**
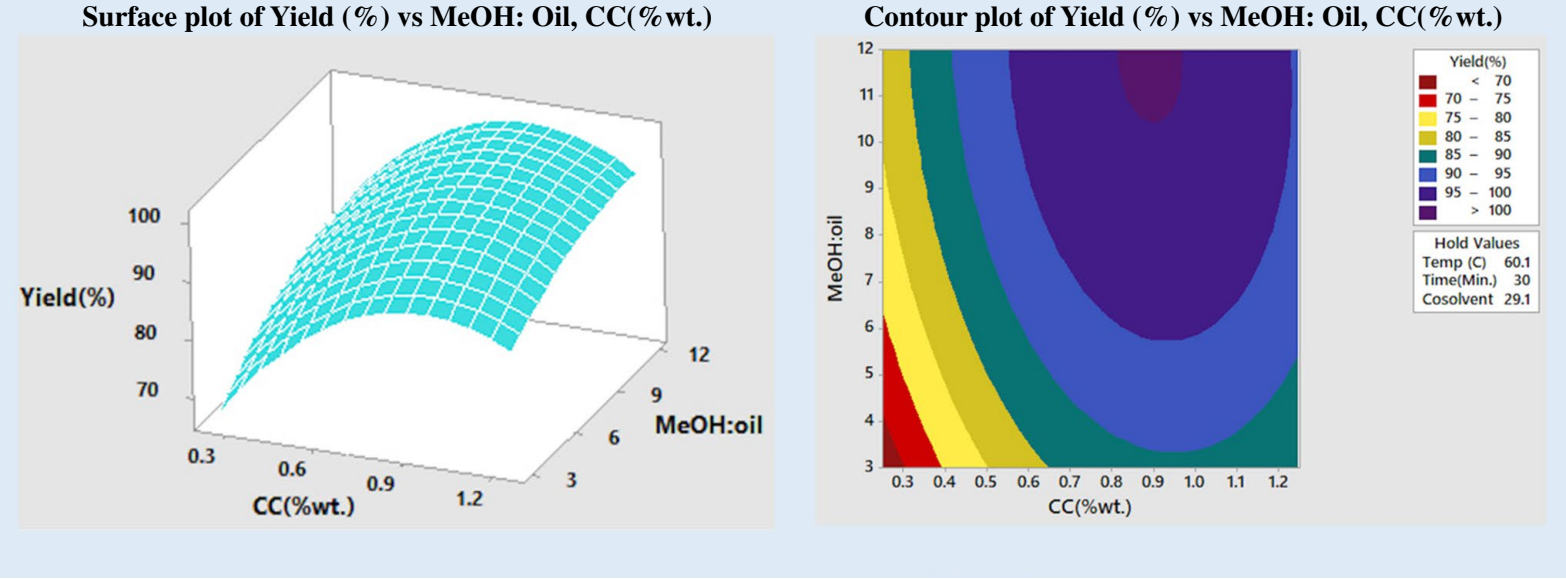
Impact of molar ratio and catalyst on yield

**Fig. 6:**
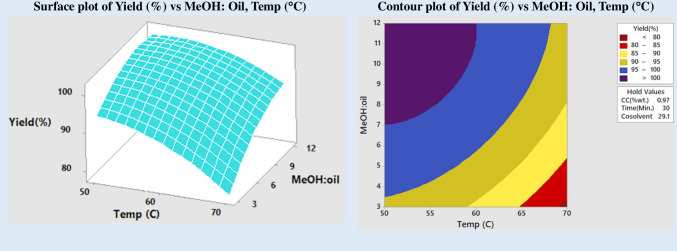
Impact of molar ratio and temperature on yield

In Fig. 5 RSM and contour plot, the yield of FAME is shown as a function of the methanol:oil molar ratio and the concentration of the catalyst. This relationship can be seen in the contour plot. According to the experiment’s findings, a specific catalyst concentration resulted in the maximum yield for each different ratio of alcohol to oil. This concentration led to the highest yield. When the ratio of methanol to oil molecules was increased, the product yield continued to rise even though the catalyst concentration remained the same. In addition, the impact of temperature changes and the methanol:oil molar ratio on product yield was examined (Fig. 6). When the molar ratio of the reactants is low, it is not favorable for transesterification to take place at high temperatures; as a result, the yield of the product reduces drastically. The quantity of alcohol that was accessible has likely decreased due to evaporation, which is a realistic explanation. A larger molar ratio impacted the total product yield less when the temperature was increased. The temperature range between 50 and 600 °C produced the best results for converting triglycerides to esters, regardless of the amount of methanol in oil.

### Impact of catalyst

**Fig. 7:**
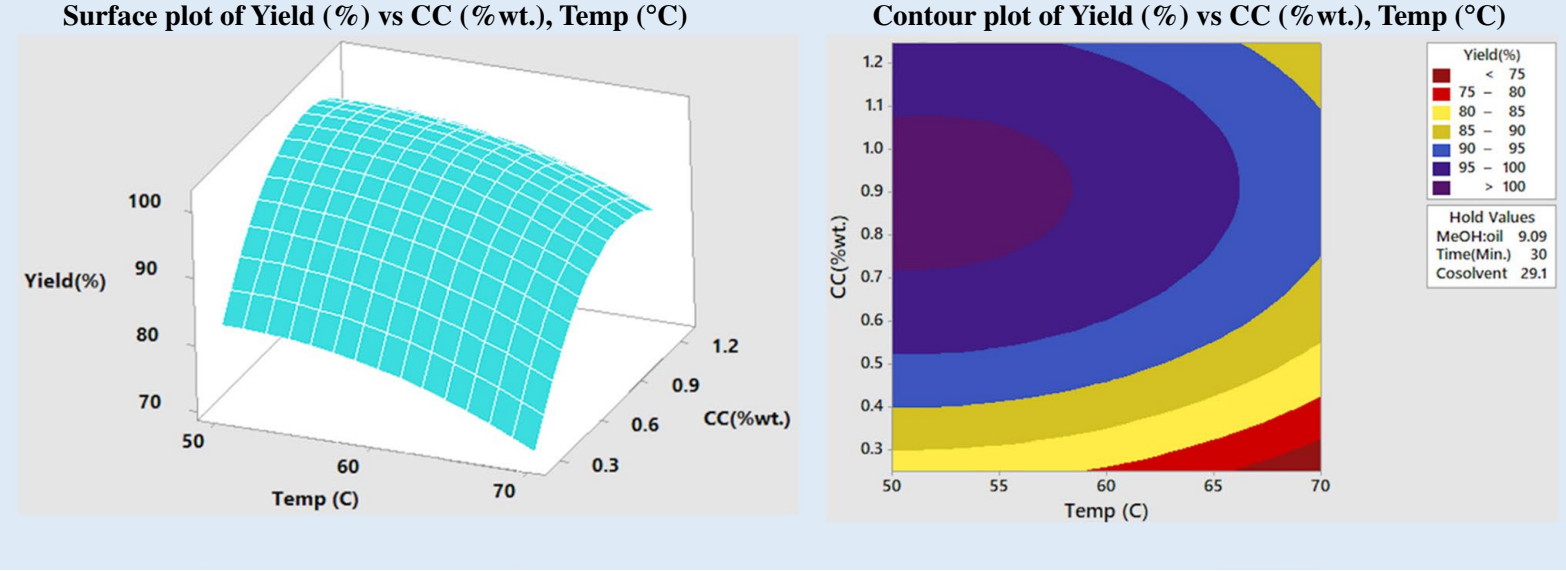
Impact of catalyst and temperature on yield

To boost the commonly sluggish transesterification process, catalysts play a crucial role. Figure 7 shows the variation of FAME yield concerning the concentration of catalyst and reaction temperature. For optimum results, keeping the alcohol-to-oil ratio, reaction time, and cosolvent at their current levels is advisable up to a catalyst concentration of 0.9 wt%; biodiesel production in the figure rises steadily, while the yield of FAME falls. This may be because the yield of FAME decreases as the concentration of the catalyst is increased above a certain point, leading to saponification and soap production (Dwivedi et al. 2022; Thangaraj et al. 2019). A high yield of FAME was predicted to be achievable at temperatures between 50 and 650 °C and catalyst concentrations of 0.5 to 0.9 wt%, as shown by the response surface model and contour map. Loss of methanol due to heating causes a decrease in product yield when the temperature exceeds the boiling point of methanol (Singh et al. 2021).

**Fig. 8:**
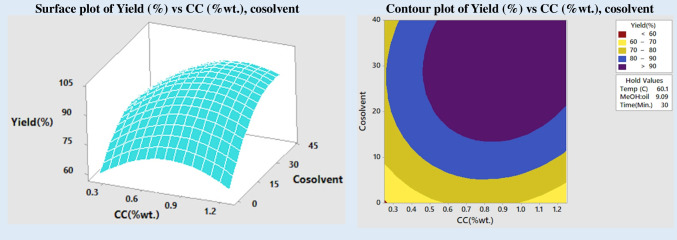
Impact of catalyst and cosolvent on yield

The effect on FAME yield is shown in Fig. 8, and it is shown as a function of simultaneously adjusting the concentration of the catalyst and the cosolvent. As was said before, the presence of a cosolvent frees up mass transfer limitations, which in turn enables a speedier transesterification process. Even at the lowest catalyst concentration (0.3 wt%), using cosolvent containing about 40 wt% allows a FAME to yield up to 80 wt%. However, the amount of cosolvent that must be utilized falls as the concentration of the catalyst rises to maintain a high level of biodiesel production.

A representation of the change in FAME yield as a function of catalyst concentration and methanol-to-oil molar ratio is shown in Fig. 5 (already described). The graph indicated that the FAME yield would rise for each catalyst concentration when the alcohol-to-oil ratio was raised. Increasing the molar ratio of alcohol to oil does not significantly improve product yield at increased catalyst concentration. According to the contour plot, a minimum alcohol-to-oil molar balance of 6:1 and a catalyst concentration of 0.8 to 0.9 wt% are needed to achieve a yield of more than 95%.

### Impact of temperature

**Fig. 9:**
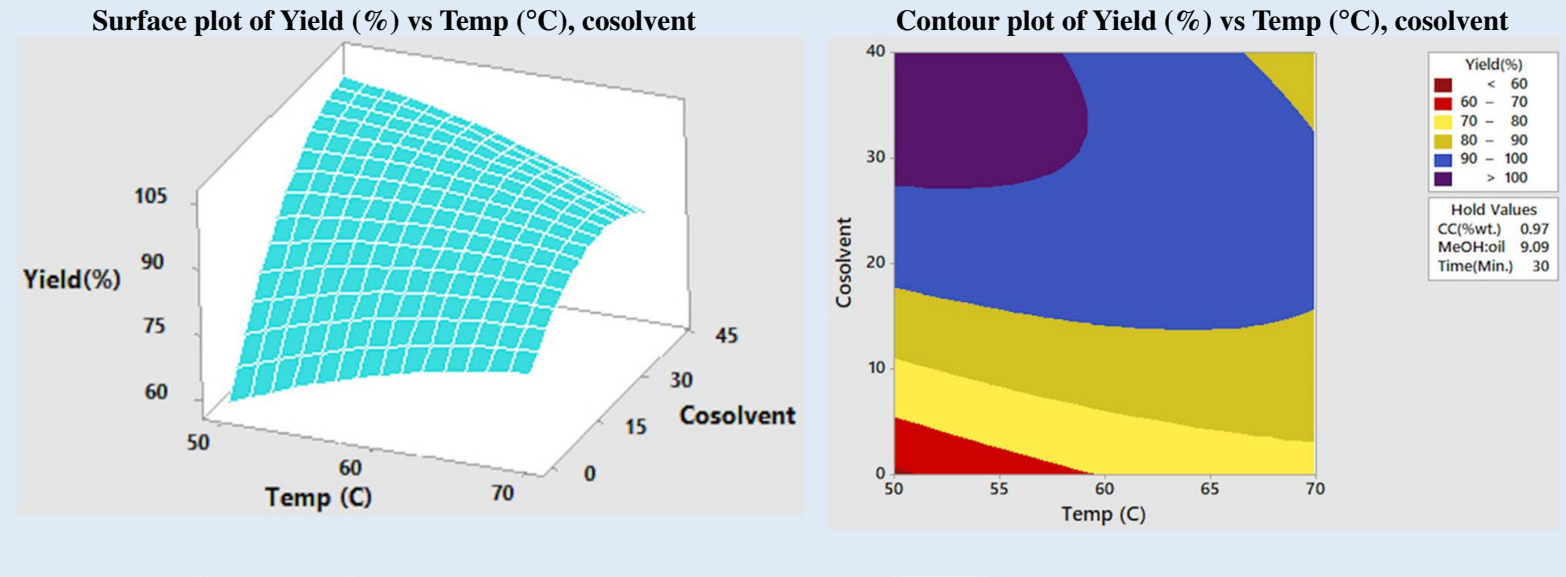
Impact of temperature and cosolvent on yield

An essential component that promotes the transesterification process is temperature. The method may be sped up by heating the oil and decreasing viscosity. Saponification of triglycerides and evaporation of alcohol at temperatures above the optimal threshold reduce the biodiesel output. This means the ideal reaction temperature is lower than the alcohol’s boiling point (Singh Pali et al. 2021). A range of temperatures (50, 55, 60, 65, and 700 °C) was used to conduct the reaction and see how changing the temperature affected transesterification. Biodiesel yield vs temperature and cosolvent concentration (Fig. 9) demonstrated that at low reaction temperature, the addition of cosolvent exhibited a notable increase in product yield. However, adding cosolvent does not significantly boost product yield when the temperature is raised. It is possible that, once again, this is due to methanol evaporation at high temperatures. So, ideally, you would aim for a temperature between 50 and 600 °C. A high FAME yield is achievable within this temperature range using a methanol-to-oil molar ratio of 6:1 to 9:1 (shown in Fig. 6). Excessive methanol addition reduces biodiesel output, raises production costs, and makes separating alcohol and glycerol more difficult.

### Impact of cosolvent

Including cosolvent in the reaction mixture enhances both the pace of the transesterification process and the amount of biodiesel that may be produced because it makes the layer of immiscible oil and alcohol more easily miscible. Even at the stoichiometric ratio of alcohol to oil, the addition of cosolvent resulted in a reasonable yield, as was previously mentioned. The FAME yield rose proportionally with the quantity of cosolvent used. It turned out that 30 wt% of cosolvent was the sweet spot. The production of esters is decreased when biodiesel and glycerol are in the same phase, which occurs when too much cosolvent is added (Mahesh et al. 2021; Torrezan et al. 2020).

Earlier, results covered how changing one or both of the cosolvent and catalyst concentrations simultaneously affects FAME production (Fig. 8). Results showed that raising the concentrations of the cosolvent and catalyst improved FAME production. At each catalyst concentration, the addition of cosolvent increased product yield at 0.6 wt% of catalyst and a minimum cosolvent concentration of 20 wt%, and yields of higher than 90% were obtained. Ester yields were lower for higher catalyst concentrations than for lower ones, regardless of the cosolvent concentration.

## Experimental tests and validations

The effort to optimize is required to raise the amount of biodiesel produced. The final value of desirability was determined to be 1. The enhanced outcomes will need to be tested and verified as a consequence. The experimental test was carried out using the currently configured environment, ensuring the RSM optimizer’s input variables were correctly set up. The RSM model was used to make predictions, and the observed values from these trials were compared to those predictions (Sathyanarayanan et al. 2022; Shobana Bai 2023). The error obtained from this comparison was then determined (Table 5). The predicted values of the output variables were discovered to be considerably different from the experiment’s outcomes. The error margin for the manufacturing yield is just 0.53%, which is a relatively small amount (Joseph Shobana Bai et al. 2023).

**Table 5:** Validation of output variables

Yield at temperature 60.1 °C, catalyst concentration 0.97 wt%, MeOH:oil ratio 9.09, time 30 min using 29.1% cosolvent			
	Predicted value (RSM)	Experimental value	Error (%)
Yield (%)	99.0253	98.5%	0.53

## Conclusion

The results of the study confirm that mass transfer restrictions between the waste fish oil and alcohol phases are overcome in an ultrasound-assisted process and the viability of the transesterification process is further improved with the addition of cosolvent. The study provides a suitable methodology to produce a high FAME yield from waste fish fat at optimized reaction conditions. The inclusion of cosolvent led to the economization of the process as the consumption of alcohol, oil, and reaction time was drastically reduced along with a satisfactory yield of biodiesel. The choice of petro-diesel as a cosolvent also had another positive aspect that it need not be removed from the final product and could be used as a blended fuel. In the future, the method may be further investigated to study the transesterification of other inexpensive and inedible oil varieties to produce more affordable fuel. Further, the method can be utilized to investigate the reaction with environment-friendly heterogeneous catalysts like biomass-based heterogeneous catalysts.

## Data Availability

The datasets used and/or analyzed during the current study are available from the corresponding author upon reasonable request.
